# Treatment guidelines and early loss from care for people living with HIV in Cape Town, South Africa: A retrospective cohort study

**DOI:** 10.1371/journal.pmed.1002434

**Published:** 2017-11-14

**Authors:** Ingrid T. Katz, Richard Kaplan, Garrett Fitzmaurice, Dominick Leone, David R. Bangsberg, Linda-Gail Bekker, Catherine Orrell

**Affiliations:** 1 Department of Medicine, Brigham and Women’s Hospital, Boston, Massachusetts, United States of America; 2 Center for Global Health, Massachusetts General Hospital, Boston, Massachusetts, United States of America; 3 Harvard Medical School, Boston, Massachusetts, United States of America; 4 Desmond Tutu HIV Centre, University of Cape Town Medical School, Cape Town, South Africa; 5 Department of Biostatistics, Harvard T.H. Chan School of Public Health, Boston, Massachusetts, United States of America; 6 Laboratory for Psychiatric Biostatistics, McLean Hospital, Belmont, Massachusetts, United States of America; 7 Ragon Institute of MGH, MIT and Harvard, Boston, Massachusetts, United States of America; 8 Oregon Health & Science University–Portland State University School of Public Health, Portland, Oregon, United States of America; San Francisco General Hospital, UNITED STATES

## Abstract

**Background:**

South Africa has undergone multiple expansions in antiretroviral therapy (ART) eligibility from an initial CD4^+^ threshold of ≤200 cells/μl to providing ART for all people living with HIV (PLWH) as of September 2016. We evaluated the association of programmatic changes in ART eligibility with loss from care, both prior to ART initiation and within the first 16 weeks of starting treatment, during a period of programmatic expansion to ART treatment at CD4^+^ ≤ 350 cells/μl.

**Methods and findings:**

We performed a retrospective cohort study of 4,025 treatment-eligible, non-pregnant PLWH accessing care in a community health center in Gugulethu Township affiliated with the Desmond Tutu HIV Centre in Cape Town. The median age of participants was 34 years (IQR 28–41 years), almost 62% were female, and the median CD4^+^ count was 173 cells/μl (IQR 92–254 cells/μl). Participants were stratified into 2 cohorts: an early cohort, enrolled into care at the health center from 1 January 2009 to 31 August 2011, when guidelines mandated that ART initiation required CD4^+^ ≤ 200 cells/μl, pregnancy, advanced clinical symptoms (World Health Organization [WHO] stage 4), or comorbidity (active tuberculosis); and a later cohort, enrolled into care from 1 September 2011 to 31 December 2013, when the treatment threshold had been expanded to CD4^+^ ≤ 350 cells/μl. Demographic and clinical factors were compared before and after the policy change using chi-squared tests to identify potentially confounding covariates, and logistic regression models were used to estimate the risk of pre-treatment (pre-ART) loss from care and early loss within the first 16 weeks on treatment, adjusting for age, baseline CD4^+^, and WHO stage. Compared with participants in the later cohort, participants in the earlier cohort had significantly more advanced disease: median CD4^+^ 146 cells/μl versus 214 cells/μl (*p <* 0.001), 61.1% WHO stage 3/4 disease versus 42.8% (*p <* 0.001), and pre-ART mortality of 34.2% versus 16.7% (*p <* 0.001). In total, 385 ART-eligible PLWH (9.6%) failed to initiate ART, of whom 25.7% died before ever starting treatment. Of the 3,640 people who started treatment, 58 (1.6%) died within the first 16 weeks in care, and an additional 644 (17.7%) were lost from care within 16 weeks of starting ART. PLWH who did start treatment in the later cohort were significantly more likely to discontinue care in <16 weeks (19.8% versus 15.8%, *p* = 0.002). After controlling for baseline CD4^+^, WHO stage, and age, this effect remained significant (adjusted odds ratio [aOR] = 1.30, 95% CI 1.09–1.55). As such, it remains unclear if early attrition from care was due to a “healthy cohort” effect or to overcrowding as programs expanded to accommodate the broader guidelines for treatment. Our findings were limited by a lack of generalizability (given that these data were from a single high-volume site where testing and treatment were available) and an inability to formally investigate the effect of crowding on the main outcome.

**Conclusions:**

Over one-quarter of this ART-eligible cohort did not achieve the long-term benefits of treatment due to early mortality, ART non-initiation, or early ART discontinuation. Those who started treatment in the later cohort appeared to be more likely to discontinue care early, and this outcome appeared to be independent of CD4^+^ count or WHO stage. Future interventions should focus on those most at risk for early loss from care as programs continue to expand in South Africa.

## Abstract 2

In a retrospective cohort study, Ingrid Katz and colleagues report on the continuity of care for people with HIV in Gugulethu Township, South Africa

## Author summary

**Why was this study done?:**

- Global HIV treatment initiatives have focused on increasing access to antiretroviral therapy, with the goal of creating an “AIDS-free generation.”
- In line with guidelines put forth by the World Health Organization, the focus is on starting newly diagnosed people on immediate treatment and maintaining long-term retention in care.
- There is growing evidence, however, that treatment availability alone is insufficient to stop the spread of the disease.
- In countries where HIV is hyperendemic such as South Africa, it remains unclear if expanding treatment eligibility to all individuals living with HIV will have an impact on patients’ engagement in care and early retention on treatment.
- We performed this study in a large, urban community HIV treatment site in Cape Town to understand the association of South Africa’s expanding treatment program with pre-treatment loss from care and early loss from care during treatment.

**What did the researchers do and find?:**

- We performed a retrospective study of 2 cohorts (which included over 4,000 treatment-eligible people living with HIV in total) who entered care at a community health center in Cape Town: an early cohort, enrolled into care from January 2009 to August 2011, when guidelines mandated that ART initiation required a CD4^+^ cell count of ≤200 cells/μl, pregnancy, advanced clinical symptoms (World Health Organization stage 4), or comorbidity (active tuberculosis), and a later cohort, enrolled September 2011 to December 2013, when the treatment threshold had been expanded to CD4^+^ ≤ 350 cells/μl.
- We determined the risk of pre-treatment and early losses (<16 weeks on treatment) from care over time, specifically assessing the association of guideline changes that expanded treatment eligibility with these losses from care.
- We found that almost 10% of the population failed to initiate treatment within 16 weeks of first learning their eligibility, and among those, over one-quarter died prior to starting treatment.
- Among those who started treatment, over 17% had stopped accessing treatment within 16 weeks of starting.

**What do these findings mean?:**

- Over one-quarter of this treatment-eligible cohort never achieved the long-term benefits of treatment due to early mortality, treatment non-initiation, or early loss from care.
- People living with HIV who presented for care during an era of expanded treatment availability were significantly more likely to discontinue care early in their treatment; however, this did not appear to be related to their health status.
- We hypothesize that this finding may reflect larger programmatic trends towards higher volume care in settings that may not have been able to proportionally expand access to healthcare providers, resulting in clinic crowding.

## Introduction

South Africa has the world’s largest HIV epidemic, which has been met with an ever expanding and increasingly robust response since 2004, enabling the development of the single biggest antiretroviral therapy (ART) program globally. There are now over 3 million people on treatment in South Africa, which represents roughly half of the people living with HIV (PLWH) in the country [1,2]. The expansion in treatment availability, first ushered in by the US President’s Emergency Plan for AIDS Relief (PEPFAR) and the Global Fund and its partners [3,4] and now predominantly run through a governmental response, has increased the availability of ART for healthier PLWH [5,6].

South Africa has undergone multiple expansions in ART eligibility, with an increasing immunological threshold for ART initiation, from a CD4^+^ threshold of ≤200 cells/μl at the start of the treatment program to providing ART for all PLWH as of September 2016. The expansion of ART eligibility has resulted in a larger number of individuals being screened for treatment. Simultaneously, funding has shifted from programs primarily funded through external donors, such as PEPFAR and the Global Fund, to clinics run by the South African Department of Health (DOH). This has resulted in transitions from centralized, physician-managed programs to more decentralized, nurse-managed clinics [7–10].

While guidelines have shifted to expand earlier access to ART, it is unknown whether the expansion of treatment eligibility and availability has had an impact on patients’ engagement in care and early retention on treatment. This is particularly critical in the context of a new era of “test and treat” that has shifted the care cascade to be more focused on earlier ART initiation and durable retention [11]. Prior research in the earliest phases of treatment availability in South Africa showed that risk of loss to follow-up and virological failure both increased over successive calendar periods as the number of patients per clinic provider increased markedly over time [5]. An individual site in Durban undergoing a rapid transfer of care from a robust PEPFAR-funded program to community-based clinics reported loss of up to 20% of patients, who may have experienced a treatment interruption [12]. Similarly, a study of over 5,000 patients transitioning care from a physician-managed clinic to a nurse-managed program showed a significantly higher rate of loss to follow-up among patients who were down-referred [13].

We performed a retrospective cohort analysis of data from a large, urban community HIV treatment site in Cape Town to assess the association of South Africa’s HIV treatment eligibility guidelines with pre-ART attrition and early loss from care (<16 weeks). We hypothesized that increasing the CD4^+^ threshold to access ART would increase pre-treatment and early losses. The rationale for this hypothesis was that there could be a healthy cohort effect and/or that the programmatic shift to expanded treatment eligibility could result in a higher patient to nurse ratio, resulting in a crowding effect.

## Methods

### Ethics statement

Data collection on this cohort was approved by the research ethics committee of the University of Cape Town and the Partners HealthCare institutional review board, and patients gave written informed consent to have data collected anonymously for research purposes.

### Treatment cohort

This cohort of PLWH accessing care in a DOH community health center in Gugulethu Township, a poor peri-urban area within Cape Town, South Africa, has been previously well characterized and is affiliated with the Desmond Tutu HIV Centre [14,15]. Treatment was made available at this site starting in September 2002, and as of 2013, over 5,257 patients were in care. While patients were not transitioned from this site during expansions in ART eligibility, there was a shift in funding starting in 2009 that resulted in an overall decrease in the number of physicians on site, while increasing the number of nurses providing care. Treatment is provided to patients free of charge. Patients have routine clinical assessments every 2 weeks prior to ART (the standard of care during this period required 8 clinic visits for patients initiating treatment) and again after 4, 8, and 16 weeks of treatment, and 16-weekly thereafter. CD4^+^ cell count testing is performed at baseline, and HIV-1 viral load is checked at 16 weeks after initiating ART.

Provision of patient care is supported by peer counselors, most of whom are living with HIV and are in care themselves [16]. Each new patient enrolling into the clinic is allocated to a peer counselor living in the same area. Through group sessions and individual home visits, patients are educated about the need for medication adherence and provided with counseling support.

### Study design

While no prospective protocol was published or registered for this observational study, we adhered to an analysis plan that was developed in advance of our study (S2 Text). Specifically, data were abstracted retrospectively on all participants on ART at the Gugulethu clinic from electronic health data collected during routine care, from first clinic access through 16 weeks after ART initiation. These data included clinical variables, treatment outcomes (including death, loss to follow-up, and transfer out), ART regimens, and laboratory data derived from patient notes and pharmacy and laboratory records. ART-naïve patients aged ≥18 years who were eligible for treatment and enrolled in this cohort between 1 January 2009 and 31 December 2013 were eligible for this analysis. Women who were pregnant were excluded from this dataset. All data that were available for analysis that met the inclusion and exclusion criteria were used. The inclusion/exclusion criteria and statistical analyses for the study were established at the outset and were not changed.

### Definitions of outcomes

Our outcomes were defined at the outset and were based on prior studies that we and others have published. Specifically, we defined “early mortality” as death from all causes prior to starting ART or death within the first 16 weeks on treatment. Pre-ART loss from care was defined as attrition between the time of learning ART eligibility and starting treatment. Early loss from care was defined as early discontinuation of treatment (within the first 16 weeks on ART). We used World Health Organization (WHO) clinical staging and immunological classification of HIV infection to assess disease status. The scale was developed in 1990 and is used only once an HIV infection has been established through a blood test [17].

### Data collection and analysis

Data were abstracted from electronic records and paper charts and included baseline CD4^+^, age at referral, WHO stage, decision-making regarding ART initiation, and early treatment outcome (up to 16 weeks on ART). WHO stage was used as a proxy measure of baseline disease severity, where those with stage 1 are predominantly asymptomatic and those with stage 4 demonstrate more pronounced symptoms. Analyses were retrospective, and treatment discontinuation was confirmed through patient tracking involving up to 3 home visits if a patient had failed to attend the clinic for ≥12 weeks and had not been traced to another regional treatment center. Participants were examined in the context of an early cohort (patients enrolled into care at the clinic between 1 January 2009 and 31 August 2011), during which time the threshold for ART initiation was CD4^+^ ≤ 200 cells/μl, and a later cohort (patients enrolled into care between 1 September 2011 and 31 December 2013), when the treatment threshold had been expanded to CD4^+^ ≤ 350 cells/μl.

Demographic and clinical factors were compared in a bivariate analysis of before and after the policy change using chi-squared tests to identify potentially confounding covariates. Baseline age was calculated from date of birth, if available, and entry into the clinical cohort. In the bivariate analysis, CD4^+^ cell count among the earlier cohort was compared to that of those entering in the later cohort, using a Wilcoxon rank sum test. In the logistic regression models, CD4^+^ cell count was dichotomized to >200 and ≤200 based on the clinical definition of an AIDS diagnosis. We used *p <* 0.20 to identify any potential confounders, and logistic regression models were then used to estimate the adjusted risk of early loss (<16 weeks) from care controlling for age, baseline CD4^+^ cell count, and WHO stage. Multiple logistic regression was used to estimate the risk of early loss from care, pre- and post-ART initiation, adjusting for relevant baseline covariates, including calendar period of enrollment, which was included as a key variable of interest in this model. Post hoc relative goodness of fit of the logistic model was verified using a log-likelihood ratio to estimate a chi-squared value. Final models were checked using standard regression diagnostics for logistic regression. Wald confidence limits were calculated for all multivariate models. All statistical tests were 2-sided at alpha of 0.05. SAS statistical software, version 9.4, was used for all analyses (SAS Institute, Cary, North Carolina).

## Results

In all, 4,025 ART-eligible PLWH who were referred to the treatment clinic between 1 January 2009 and 31 December 2013 were included in our sample. The median age in our population was 34 years (IQR 28–41 years) (see Table 1). Nearly 62% were female, and the median CD4^+^ count was 173 cells/μl (IQR 92–254 cells/μl). Overall, individuals in the earlier cohort had significantly more advanced disease than those in the later cohort, with lower CD4^+^ counts at the time of ART initiation (146 cells/μl versus 214 cells/μl, respectively, *p <* 0.001), and a larger percentage were classified as having a higher WHO stage (61.1% with stage 3/4 versus 42.8%, respectively, *p <* 0.001).

**Table 1:** Baseline characteristics of all participants by qualification period.

Characteristic	Total cohort (*n* = 4,025)	Early cohort^1^ (*n* = 2,123)	Later cohort^2^ (*n* = 1,902)	*p*-Value^3^
**Age, years**^4^	34 (28–41)	35 (29–42)	34 (28–41)	**0.01**
**Female sex**^5^	2,489 (61.9%)	1,299 (61.2%)	1,190 (62.6%)	0.36
**WHO stage**^6^				<**0.001**
1	1,144 (28.5%)	436 (20.6%)	708 (37.3%)	
2	764 (19.0%)	387 (18.3%)	377 (19.9%)	
3	1,624 (40.4%)	978 (46.1%)	646 (34.0%)	
4	484 (12.1%)	318 (15.0%)	166 (8.8%)	
**Baseline CD4**^**+**^, **cells/μl**	173 (92–254)	146 (75–215)	214 (119–298)	<**0.001**

Data are given as median (IQR) or number (percent).

^1^Early cohort is the period prior to the 2011 policy change, during which time the threshold for ART initiation was CD4^+^ ≤ 200 cells/μl.

^2^Later cohort is the period after the 2011 policy change, during which time the threshold for ART initiation was CD4^+^ ≤ 350 cells/μl.

^3^A Wilcoxon rank sum test was used to estimate the *p*-value comparing median age between the 2 cohorts; all other *p*-values were generated using a chi-squared test of independence. Bold indicates a statistically significant difference.

^4^Missing age: early cohort, *n* = 1; later cohort, *n* = 1.

^5^Missing sex: early cohort, *n* = 0; later cohort, *n* = 1.

^6^Missing stage: early cohort, *n* = 4; later cohort, *n* = 5.

Ninety percent (*n* = 3,640) of the population initiated ART within 16 weeks of first entering the clinic, per pharmacy records (see Table 2). There was no significant difference in the percentage of the population who initiated ART within 16 weeks between the earlier and later cohorts (90.6% versus 90.2%, *p* = 0.7). Of the 9.6% (*n* = 385) who did not start ART, 25.7% died prior to starting treatment. The rate of pre-ART death in the earlier cohort was twice that of the later cohort (34.2% versus 16.7%, *p <* 0.001).

**Table 2:** Early loss from care and mortality in Cape Town, South Africa.

Outcome	Total cohort (*n* = 4,025)	Early cohort^1^ (*n* = 2,123)	Later cohort^2^ (*n* = 1,902)	*p*-Value^3^
**Initiated ART**	3,640/4,025 (90.4%)	1,924/2,123 (90.6%)	1,716/1,902 (90.2%)	0.7
In care through 16 weeks	2,938/3,640 (80.7%)	1,577/1,924 (82.0%)	1,361/1,716 (79.3%)	**0.05**
Stopped care <16 weeks	644/3,640 (17.7%)	305/1,924 (15.9%)	339/1,716 (19.8%)	**0.002**
Died ≤16 weeks	58/3,640 (1.6%)	42/1,924 (2.2%)	16/1,716 (0.9%)	**0.004**
**No ART initiated**	385/4,025 (9.6%)	199/2,123 (9.4%)	186/1,902 (9.8%)	0.7
Died pre-ART	99/385 (25.7%)	68/199 (34.2%)	31/186 (16.7%)	<**0.001**

^1^Early cohort is the period prior to the 2011 policy change, during which time the threshold for ART initiation was CD4^+^ ≤ 200 cells/μl.

^2^Later cohort is the period after the 2011 policy change, during which time the threshold for ART initiation was CD4^+^ ≤ 350 cells/μl.

^3^*p*-Values were generated using a chi-squared test of independence. Bold indicates a statistically significant difference.

Among the cohort who initiated treatment, 17.7% stopped accessing treatment within 16 weeks of ART initiation, and 1.6% died within the first 16 weeks. ART-eligible individuals in the later cohort were significantly more likely to discontinue care <16 weeks into treatment compared to those initiating treatment prior to 31 August 2011 (19.8% versus 15.8%, odds ratio [OR] = 1.32, *p* = 0.002). After controlling for baseline CD4^+^, WHO stage, and age, this effect remained significant (adjusted OR [aOR] = 1.30, 95% CI 1.09–1.55). When the analysis was restricted to only the individuals who would have qualified for ART in either cohort (CD4^+^ ≤ 200 cells/μl), the difference in early ART discontinuation remained significant (aOR = 1.34, 95% CI 1.06–1.67).

**Fig 1:**
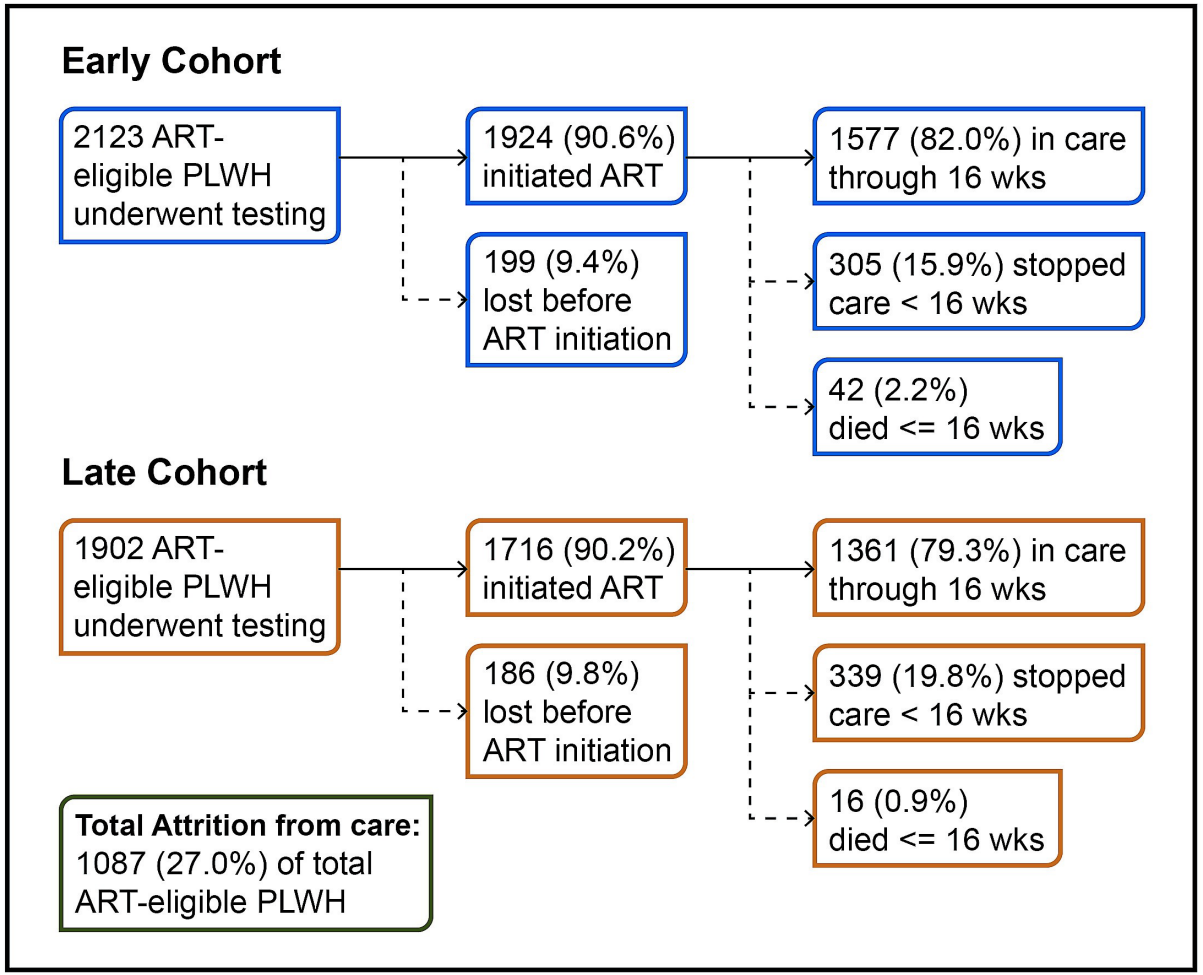
Attrition in the care cascade. PLWH, people living with HIV.

Fig 1 shows attrition across the cascade from the pre-ART period through to 16 weeks on ART in both the early and later cohorts. Across the full cohort, 157 (3.9%) ART-eligible PLWH died, and 930 (23.1%) were lost from care prior to ART initiation or within the first 16 weeks of starting treatment. This resulted in a total combined early loss of 1,087 (27.0%) ART-eligible PLWH. Over the 5 years of the study period, the total number of people entering care increased over 2-fold, from 776 entering care in 2009 to 1,506 entering care in 2013. During this time, the Gugulethu clinic transitioned from 5 doctors and 5 nurses in 2009 to 3 doctors and 7 nurses in 2013. The standard of care during this period required 8 clinic visits for patients initiating treatment.

## Discussion

In this cohort study, we assessed early losses from care over a 5-year period as South Africa was expanding its ART eligibility, and shifting from a centralized HIV treatment program with a high number of medical doctors, funded through PEPFAR, the Global Fund, and other partners, to a more decentralized, nurse-led system, supported largely through the South African government. Overall, we found that over one-quarter of this well-established ART-eligible cohort never achieved the long-term benefits of treatment and viral load suppression due to early mortality, failure to start ART, or ART discontinuation <16 weeks from the time of initiation. Patients who entered care in the later cohort were significantly more likely to discontinue treatment early.

Estimates of pre-ART attrition from care and early loss from care on ART among ART-eligible PLWH have consistently been estimated at 20%–30% of patients in South Africa [18–21]. What has remained less clear is the impact of guideline changes and shifting delivery systems on attrition and early loss over time. In 2013, WHO published consolidated guidelines on the use of antiretroviral drugs for HIV treatment and prevention across all age groups and populations, that were then revised based on new scientific evidence in 2015 [22]. WHO set forth these guidelines to increase treatment CD4^+^ thresholds, with the explicit goal of reducing transmission between sexual partners [23] and providing health benefits for patients taking treatment [24–26].

As the South African government has moved to incorporate these recommendations, expanding treatment to all PLWH, more people have become eligible to start ART. However, challenges remain in both ART initiation and retention in care, resulting in the failure of these strategies to achieve the expected benefits for the individual, let alone at a population level [27]. Recent research by Haber et al. showed that in a large population of PLWH in South Africa, the early stages of the cascade were the most vulnerable to losses [28]. South Africa has faced the added challenge of transitioning from a well-resourced HIV care program supported by donors to a decentralized system of care though a vast network of public health clinics, while expanding enrollment into ART programs throughout the country.

The impact of policy-level changes on treatment outcomes remains uncertain. In this study, the rate of early loss continued to rise throughout the study period. While the later cohort was significantly healthier than the earlier cohort, the increase in loss from care persisted in adjusted analyses and thus did not appear to reflect differences in CD4 count and WHO stage. We believe this trend may reflect large programmatic shifts in care and clinic crowding, as efforts have continued to expand to accommodate millions more PLWH into treatment programs despite constrained resources and a limited number of healthcare providers [3,29]. In addition, our prior qualitative research [30], along with prior research conducted by Fox et al. [31] and Duff et al. [32], suggests that perceptions of health and illness may be a strong driver of ART decision-making, and that these perceptions may not always be correlated with actual CD4^+^ counts [33]. As such, the goal of getting patients onto treatment earlier may remain a challenge if PLWH perceive themselves to be healthy, especially in settings where clinic crowding remains significant.

Our data have several limitations and a number of strengths. First, we are limited by the fact that these data were accumulated at a single high-volume site. Therefore, it is unclear if these findings are generalizable. Despite this, our findings are consistent with data from other multi-site, large cohorts [27,28,34]. Second, despite our access to staffing numbers, we are unable to formally investigate whether a crowding effect was the true cause of higher rates of loss from care in the later cohort. Third, our sample cannot account for patients who have potentially left the area and accessed care in other provinces. While this is a challenge consistently noted in previous research [35,36], this clinic utilized a robust tracking system when patients did not return for treatment, including active tracing of patients who missed clinic visits through home visits by community care workers. Finally, these data were collected from a site where treatment and testing were available. As such, it remains unclear if the linkage rates observed would be as high if the site was a stand-alone testing site where patients then had to link to a new site for care.

In conclusion, over one-quarter of this well-established ART-eligible cohort did not achieve the long-term benefits of treatment due to early mortality, failure to start ART, or ART discontinuation <16 weeks in care. Early ART discontinuation, which appeared to be independent of CD4^+^ count or WHO stage, likely reflected larger programmatic trends towards higher volume treatment centers that result in clinic crowding. Future interventions should focus on those most at risk for pre-ART attrition and early loss from care as programs continue to expand in an era of treatment for all in South Africa.

## Supporting information

S1 Text STROBE checklist. (DOCX)

S2 Text Analysis plan. (DOCX)

## Abbreviations

aOR: adjusted odds ratio
ART: antiretroviral therapy
DOH: Department of Health
OR: odds ratio
PEPFAR: US President’s Emergency Plan for AIDS Relief
PLWH: people living with HIV
WHO: World Health Organization
