# Supplementary material for: Treatment guidelines and early loss from care for people living with HIV in Cape Town, South Africa: A retrospective cohort study
Source: PLoS Med. 2017 Nov 14;14(11):e1002434. doi: 10.1371/journal.pmed.1002434 (PMC5685472; doi:10.1371/journal.pmed.1002434)
Supplement: S1 Text — (DOCX) [file pmed.1002434.s001.docx]

STROBE Statement—checklist of items that should be included in reports of observational studies

|  | | Item No. | Recommendation | | | Section | Relevant text from manuscript |  |
| --- | --- | --- | --- | --- | --- | --- | --- | --- |
| **Title and abstract** | | 1 | (*a*) Indicate the study’s design with a commonly used term in the title or the abstract | | | Abstract, Methods and Findings | “We performed a retrospective cohort study of 4025 treatment-eligible people living with HIV (PLWH) accessing care in a Community Health Centre in Gugulethu Township, affiliated with the Desmond Tutu HIV Centre in Cape Town.” |  |
|  |  |  | (*b*) Provide in the abstract an informative and balanced summary of what was done and what was found | | | Abstract, Methods and Findings | “Participants were stratified into two cohorts: an early cohort (from January 2009 to August 2011) when guidelines mandated ART initiation required a CD4^+^<200 cells/μL, pregnancy, advanced clinical symptoms (World Health Organization [WHO] stage IV), or co-morbidities (active tuberculosis); and a later cohort (September, 2011 to December, 2013) when the treatment threshold expanded to CD4^+^<350 cells/μL.Demographic and clinical factors were compared pre-and-post policy change using chi-square to identify potentially confounding covariates, and logistic regression models were used to estimate the risk of pre-treatment losses (pre-ART) and early losses within the first16 weeks on treatment, adjusting for age, baseline CD4, and WHO stage. As compared with participants in the later cohort, participants in the earlier cohort had significantly more advanced disease: median CD4^+^ 146 cells/μL vs. 214 cells/μL (p<0.001); 61.1% WHO stages III/IV vs. 42.8% (p<0.001); and pre-ART mortality of 34.2% vs. 16.7% (p<0.001) respectively. In total, 385 ART-eligible PLWH (9.6%) failed to initiate ART, of whom 25.7% died before ever entering care. Of the 3640 people who started treatment, 58 (1.6%) died within the first 16 weeks in care, and an additional 644 (17.7%) were lost within 16 weeks of starting ART. PLWH who did start treatment in the later cohort were significantly more likely to discontinue care in <16 weeks (19.8% vs. 15.8%, p=.002). After controlling for baseline CD4^+^, WHO stage, and age, this effect remained significant (Adjusted Odds Ratio (aOR)=1.30, 95%CI: 1.09–1.55).” |  |
| Introduction | | | | | | |  |  |
| Background/rationale | | 2 | Explain the scientific background and rationale for the investigation being reported | | | Introduction, Paragraphs 2 and 4 | “South Africa has undergone multiple expansions in antiretroviral (ART) eligibility from an initial CD4^+^ threshold of <200 cells/μL to providing ART for all people living with HIV (PLWH) as of September, 2016…”  “We performed a retrospective cohort analysis of data from a large urban community HIV-treatment site in Cape Town to assess the association of South Africa’s HIV treatment eligibility guidelines with pre-ART attrition with early losses from care (<16 weeks).” |  |
| Objectives | | 3 | State specific objectives, including any prespecified hypotheses | | | Introduction, Paragraph 4 | “We hypothesized that increasing the CD4^+^ threshold to access ART would increase pre-treatment and early losses. The rational for this hypothesis is due to healthy cohort effects, and/or programmatic shifts resulting in expanded clinics with higher patient to nurse ratio, resulting in a “crowding effect.’” |  |
| Methods | | | | | | |  |  |
| Study design | | 4 | Present key elements of study design early in the paper | | | Methods, Paragraph 4 (Study Design Section) | “Data were abstracted retrospectively on all participants on ART at Gugulethu using electronic health data collected during routine care, from first clinic access through 16 weeks after ART initiation. These data included: clinical variables, treatment outcomes (including death, loss to follow-up, and transfer out), ART regimens, and laboratory data derived from patient notes, pharmacy and laboratory records.” |  |
| Setting | | 5 | Describe the setting, locations, and relevant dates, including periods of recruitment, exposure, follow-up, and data collection | | | Methods, Paragraph 2 (Treatment Cohort Section) and Paragraph 4 (Study Design Section) | “This cohort of PLWH accessing care in a DOH Community Health Centre in Gugulethu Township, a poor peri-urban area within Cape Town, South Africa, has been previously well characterized and is affiliated with the Desmond Tutu HIV Centre.”  “ART-naïve patients aged ≥18 years who were eligible for treatment and enrolled in this cohort between January 2009 and December 2013 were eligible for this analysis. Women who were pregnant were excluded from this dataset.” |  |
| Participants | | 6 | (*a*) *Cohort study*—Give the eligibility criteria, and the sources and methods of selection of participants. Describe methods of follow-up | | | Methods, Paragraph 4 (Study Design Section) | “ART-naïve patients aged ≥18 years who were eligible for treatment and enrolled in this cohort between January 2009 and December 2013 were eligible for this analysis. Women who were pregnant were excluded from this dataset.” |  |
|  |  |  | (*b*) *Cohort study*—For matched studies, give matching criteria and number of exposed and unexposed | | |  | No matching was performed in this study-analysis. |  |
| Variables | | 7 | Clearly define all outcomes, exposures, predictors, potential confounders, and effect modifiers. Give diagnostic criteria, if applicable | | | Methods, Paragraph 5 (Definitions of Outcomes) | “We defined ‘early mortality’ as death from all causes prior to starting ART, or death within the first 16 weeks on treatment. Pre-ART losses was defined as attrition between the time of learning ART eligibility, and entering care. Early losses were defined as early discontinuation of treatment (within the first 16 weeks in care). We used World Health Organization(WHO) clinical staging and immunological classification of HIV infection to assess disease status. The scale was developed in 1990 and is used only once an HIV infection has been established through a blood test.” |  |
| Data sources/ measurement | | 8* | For each variable of interest, give sources of data and details of methods of assessment (measurement). Describe comparability of assessment methods if there is more than one group | | | Methods, Paragraph 7 (Data Collection and Analysis) | “Demographic and clinical factors were compared in a bivariate analysis of pre-and-post policy change using chi-square to identify potentially confounding covariates. Baseline age was calculated from date of birth, if available, and entry into the clinical cohort. In the bivariate analysis, CD4^+^ cell count among the earlier cohort was compared to those entering in the later cohort, using a Wilcoxon Rank Sum. In the logistic regression models, CD4^+^ cell count was dichotomized to ≥200 and <200 based on the clinical definition of an AIDS diagnosis. We used p<.20 to identify any potential confounders, and logistic regression models were then used to estimate the adjusted risk of early loss (<16 weeks) from care controlling for age, baseline CD4^+^ cell count, and WHO stage.” |  |
| Bias | | 9 | Describe any efforts to address potential sources of bias | | | Methods, Paragraph 7 (Data Collection and Analysis) | “Multiple Logistic regression was used to estimate the risk of early losses, and mortality pre- and post-ART initiation, adjusting for relevant baseline covariates, including calendar period of enrollment which was included as a key variable of interest in this model. Post-hoc, relative goodness of fit of the logistic model was verified using a log-likelihood ratio to estimate a chi-square. Final models were checked using standard regression diagnostics for logistic regression. Wald confidence limits were used for all multivariate models. All statistical tests were two-sided at alpha of 0.05.” |  |
| Study size | | 10 | Explain how the study size was arrived at | | | Methods, Paragraph 4 (Study Design Section) | “All data that was available for analysis that met the inclusion and exclusion criteria were used. “ |  |
| Quantitative variables | 11 | Explain how quantitative variables were handled in the analyses. If applicable, describe which groupings were chosen and why | | | | Methods, Paragraph 7 (Data Collection and Analysis) | “Data were abstracted from electronic records and paper charts and included baseline CD4^+^, age at referral WHO stage, decision-making regarding ART initiation, and early treatment outcome (up to 16 weeks on ART). WHO stage was used as a proxy measure of baseline disease severity, where those with stage 1 are predominantly asymptomatic and those with stage 4 demonstrate more pronounced symptoms. Analyses were retrospective, and treatment discontinuation was confirmed through patient tracking involving up to 3 home visits if a patient had failed to attend the clinic for ≥12 weeks, and had not been traced to another regional treatment center. Participants were examined in the context of an early cohort (January 2009-August 2011), during which time the threshold for ART initiation was CD4^+^<200 cells/μL, and a later cohort (September, 2011-December, 2013), when the treatment threshold expanded to CD4^+^<350 cells/μL.” |  |
| Statistical methods | 12 | (*a*) Describe all statistical methods, including those used to control for confounding | | | | Methods, Paragraph 8 (Data Collection and Analysis) | “Demographic and clinical factors were compared in a bivariate analysis of pre-and-post policy change using chi-square to identify potentially confounding covariates. Baseline age was calculated from date of birth, if available, and entry into the clinical cohort. In the bivariate analysis, CD4^+^ cell count among the earlier cohort was compared to those entering in the later cohort, using a Wilcoxon Rank Sum. In the logistic regression models, CD4^+^ cell count was dichotomized to ≥200 and <200 based on the clinical definition of an AIDS diagnosis. We used p<.20 to identify any potential confounders, and logistic regression models were then used to estimate the adjusted risk of early loss (<16 weeks) from care controlling for age, baseline CD4^+^ cell count, and WHO stage. Multiple Logistic regression was used to estimate the risk of early losses, and mortality pre- and post-ART initiation, adjusting for relevant baseline covariates, including calendar period of enrolment which was included as a key variable of interest in this model. Post-hoc, relative goodness of fit of the logistic model was verified using a log-likelihood ratio to estimate a chi-square. Final models were checked using standard regression diagnostics for logistic regression. Wald confidence limits were used for all multivariate models. All statistical tests were two-sided at alpha of 0.05.” |  |
|  |  | (*b*) Describe any methods used to examine subgroups and interactions | | | |  | (b) Subgroups were in explored in bivariate analysis to determine potential for confounding. No interactions were examined. |  |
|  |  | (*c*) Explain how missing data were addressed | | | |  | (c) Analyses were limited to non-missing data only. |  |
|  |  | (*d*) *Cohort study*—If applicable, explain how loss to follow-up was addressed  *Case-control study*—If applicable, explain how matching of cases and controls was addressed  *Cross-sectional study*—If applicable, describe analytical methods taking account of sampling strategy | | | |  | (d) Clinic staff made multiple attempts to reach patients who were lost to clinical care including home and community visits. |  |
|  |  | (*e*) Describe any sensitivity analyses | | | |  | (e) No sensitivity analyses were done. |  |
| Results | | | | | | | |  |
| Participants | 13* | (a) Report numbers of individuals at each stage of study—eg numbers potentially eligible, examined for eligibility, confirmed eligible, included in the study, completing follow-up, and analysed | | | | Results, paragraph 1 | “4025 ART-eligible PLWH who were referred to the treatment clinic between January 2009 and December 2013 were included in our sample.” |  |
|  |  | (b) Give reasons for non-participation at each stage | | | |  |  |  |
|  |  | (c) Consider use of a flow diagram | | | |  |  |  |
| Descriptive data | 14* | (a) Give characteristics of study participants (eg demographic, clinical, social) and information on exposures and potential confounders | | | | Results, paragraph 1 | “The median age in our population was 34 years (IQR: 28 years – 41 years) (see **Table 1**). Nearly 62% were female, and the median CD4^+^ was 173 cells/μL (IQR: 92 cells/μL – 254 cells/μL). Overall, individuals in the earlier cohort had significantly more advanced disease, with lower CD4^+^ counts at the time of ART initiation (146 cells/μL in the earlier cohort vs. 214 cells/μL in the later cohort, p<.0001), and a larger percentage were classified as having a higher WHO stage (61.1% with stages III and IV in the earlier cohort vs. 42.8% in the later cohort, p<.0001).” |  |
|  |  | (b) Indicate number of participants with missing data for each variable of interest | | | |  | 2 subjects missing age (1 in each subcategory of programmatic period); 1 subject missing age in later cohort; and 9 missing WHO stage (4 in earlier cohort vs 5 in the later). |  |
|  |  | (c) *Cohort study*—Summarise follow-up time (eg, average and total amount) | | | |  | This is an analysis of the first 16 weeks of clinical care at a public health clinic, therefore we did not report follow-up time. |  |
| Outcome data | 15* | *Cohort study*—Report numbers of outcome events or summary measures over time | | | | Results, paragraph 3 | “Among the cohort who initiated treatment, 17.7% had stopped accessing treatment within 16 weeks of ART initiation, and 1.6% died within the first 16 weeks. ART-eligible individuals in the later sub-cohort were significantly more likely to discontinue care <16 weeks into treatment (19.8% vs. 15.8%, OR=1.32, p=0.002).” |  |
| Main results | 16 | (*a*) Give unadjusted estimates and, if applicable, confounder-adjusted estimates and their precision (eg, 95% confidence interval). Make clear which confounders were adjusted for and why they were included | | | | Results, paragraph 4 | “Across the full cohort, 157 (3.9%) ART-eligible PLWH died, and 930 (23.1%) were lost prior to ART initiation or within the first 16 weeks of starting treatment. This resulted in a total combined early loss of 1087 (27.0%) ART-eligible PLWH. Over the five years of the cohort, the total number of people entering care increased over two-fold, from 776 entering care in 2009, to 1506 entering care in 2013. During this time, the Gugulethu clinic transitioned from five doctors and five nurses in 2009 to three doctors and seven nurses in 2013. The standard of care during this period required eight clinic-visits for patients initiating treatment.”  CD4 and WHO stage were included to test the healthy cohort hypothesis; age was included as a potential confounder based on its association with both programmatic period and early loss in bivariate analysis. |  |
|  |  | (*b*) Report category boundaries when continuous variables were categorized | | | |  | “CD4^+^ cell count was dichotomized to ≥200 and <200 based on the clinical definition of an AIDS diagnosis.” |  |
|  |  | (*c*) If relevant, consider translating estimates of relative risk into absolute risk for a meaningful time period | | | |  | N/A |  |
| Other analyses | 17 | Report other analyses done—eg analyses of subgroups and interactions, and sensitivity analyses | | | | | No additional analyses beyond those listed above |  |
| Discussion | | | | | | | | |
| Key results | 18 | Summarise key results with reference to study objectives | | | | Discussion, Paragraph 1 | “Overall, we found that over one-quarter of this well-established ART-eligible cohort never achieved the long-term benefits of treatment and viral load suppression due to early mortality, failure to start ART, or ART discontinuation < 16 weeks from the time of initiation. Patients who entered care in the later part of the cohort were significantly more likely to discontinue treatment early.” |  |
| Limitations | 19 | Discuss limitations of the study, taking into account sources of potential bias or imprecision. Discuss both direction and magnitude of any potential bias | | Discussion, Paragraph 5 | | | “Our data have several limitations and a number of strengths. We are limited by the fact that these data were accumulated at a single, high-volume site. Therefore, it is unclear if these findings are generalizable. Despite this, our findings are consistent with other multi-site, large cohort data. In addition, despite our access to staffing numbers, we are unable to formally investigate whether a crowding effect was the true cause of higher rates of losses in the later cohort. Third, our sample cannot account for populations who are highly mobile, and who have potentially established care in other provinces. While this is a challenge consistently noted in prior research [35,36], this clinic utilized a robust tracking system when patients have not returned for treatment, including active tracing of patients who have missed clinic visits through home visits by community care workers. Finally, these data were collected from a site where treatment and testing were available. As such, it remains unclear if the linkage rates observed would be as high if the site was a stand-alone testing site where patients then had to link to a new site for care.” |  |
| Interpretation | 20 | Give a cautious overall interpretation of results considering objectives, limitations, multiplicity of analyses, results from similar studies, and other relevant evidence | | | Discussion, Paragraph 6 | | “Over one-quarter of this well-established ART-eligible cohort never achieved the long-term benefits of treatment due to early mortality, failure to start ART, or ART discontinuation < 16 weeks in care. Early ART discontinuation which appeared to be independent of CD4^+^ count or WHO stage, likely reflected larger programmatic trends towards higher volume treatment centers that results in clinic “crowding.” Future interventions should focus on those most at risk for pre-ART attrition and early losses from care as programs continue to expand in an era of treatment for all in South Africa.” |  |
| Generalisability | 21 | Discuss the generalisability (external validity) of the study results | | | | | As per above, given we collected these data at a single site, it is unclear if these findings are generalizable. Despite this, our findings are consistent with other multi-site, large cohort data. |  |
| Other information | |  | | | | | | |
| Funding | 22 | Give the source of funding and the role of the funders for the present study and, if applicable, for the original study on which the present article is based | | | | | **“**Dr. Katz’s time on this manuscript was supported by the U.S. National Institute for Mental Health K23 MH097667. The content is solely the responsibility of the authors and does not necessarily represent the official views of the National Institutes of Health. The funders had no role in study design, data collection and analysis, decision to publish, or preparation of the manuscript.” |  |

*Give information separately for cases and controls in case-control studies and, if applicable, for exposed and unexposed groups in cohort and cross-sectional studies.

**Note:** An Explanation and Elaboration article discusses each checklist item and gives methodological background and published examples of transparent reporting. The STROBE checklist is best used in conjunction with this article (freely available on the Web sites of PLoS Medicine at http://www.plosmedicine.org/, Annals of Internal Medicine at http://www.annals.org/, and Epidemiology at http://www.epidem.com/). Information on the STROBE Initiative is available at www.strobe-statement.org.
