# Supplementary material for: Treatment guidelines and early loss from care for people living with HIV in Cape Town, South Africa: A retrospective cohort study
Source: PLoS Med. 2017 Nov 14;14(11):e1002434. doi: 10.1371/journal.pmed.1002434 (PMC5685472; doi:10.1371/journal.pmed.1002434)
Supplement: S2 Text — (DOCX) [file pmed.1002434.s002.DOCX]

**Adherence to Analysis Protocol**

Supporting information for:

**Early losses from care and treatment guidelines for people living with HIV in Cape Town South Africa: a retrospective cohort study**

Ingrid T Katz,*^1,2,3^ Richard Kaplan,^4^ Garrett Fitzmaurice,^5,6^ Dominick Leone,^7^ David R. Bangsberg,^8^ Linda-Gail Bekker,^4^ Catherine Orrell,^4^

^1^ Department of Medicine, Brigham and Women’s Hospital, Boston, Massachusetts, United States of America;

^2^ Massachusetts General Hospital, Center for Global Health, Boston, Massachusetts, USA;
^3^ Harvard Medical School, Boston, Massachusetts, United States of America;

^4^ Desmond Tutu HIV Centre, University of Cape Town Medical School, Cape Town, South Africa;

^5^ Department of Biostatistics, Harvard School of Public Health, Boston, Massachusetts, USA;

^6^ Laboratory for Psychiatric Biostatistics, McLean Hospital, Belmont, United States;

^7^ Ragon Institute, Massachusetts General Hospital, Massachusetts Institute of Technology, and Harvard University, Boston, United States;

^8^ Oregon Health & Science University-Portland State University School of Public Health, Portland, United States

*[ikatz2@partners.org](mailto:ikatz2@partners.org)

- No prospective protocol was published or registered for this observational study. We did, however, follow a clear analysis plan, as described in the methods section, and did not deviate from this plan.
- The inclusion/exclusion criteria for the study were established at the outset and were not changed. The study included all ART-naïve patients aged ≥18 years who were accessing care at the Department of Health Community Health Center in Gugulethu Township, South Africa. We restricted our analyses to the period between January 2009 and December 2013 to assess the impact of programmatic expansion on outcomes. Women who were pregnant were excluded from this dataset.
- Our definitions were established at the outset and were not changed. Specifically, we defined “early mortality” as death from all causes prior to starting ART, or death within the first 16 weeks on treatment. Pre-ART losses was defined as attrition between the time of learning ART eligibility, and entering care. Early losses were defined as early discontinuation of treatment (within the first 16 weeks in care). We used World Health Organization(WHO) clinical staging and immunological classification of HIV infection to assess disease status.
- The data collection plan was pre-specified from the outset and was not altered. Specifically, the data were abstracted from electronic records and paper charts and included baseline CD4^+^, age at referral WHO stage, decision-making regarding ART initiation, and early treatment outcome (up to 16 weeks on ART). WHO stage was used as a proxy measure of baseline disease severity, where those with stage 1 are predominantly asymptomatic and those with stage 4 demonstrate more pronounced symptoms.
- Statistical analyses were determined at the outset and were not changed. We planned to assess the association of South Africa’s HIV treatment eligibility guidelines with pre-ART attrition and with early losses from care (<16 weeks). We hypothesized that increasing the CD4^+^ threshold to access ART would increase pre-treatment and early losses. The rational for this hypothesis is due to healthy cohort effects, and/or programmatic shifts resulting in expanded clinics with higher patient to nurse ratio, resulting in a “crowding effect.” We also assessed mortality pre- and post-ART initiation, adjusting for relevant baseline covariates, including calendar period of enrollment.
- Our methods for calculating early losses from care pre- and post-policy change were determined at the outset and were not changed. Specifically, demographic and clinical factors were compared in a bivariate analysis of pre-and-post policy change using chi-square to identify potentially confounding covariates. Baseline age was calculated from date of birth, if available, and entry into the clinical cohort. In the bivariate analysis, CD4^+^ cell count among the earlier cohort was compared to those entering in the later cohort, using a Wilcoxon Rank Sum. In the logistic regression models, CD4^+^ cell count was dichotomized to ≥200 and <200 based on the clinical definition of an AIDS diagnosis. We used p<.20 to identify any potential confounders, and logistic regression models were then used to estimate the adjusted risk of early loss (<16 weeks) from care controlling for age, baseline CD4^+^ cell count, and WHO stage. Multiple Logistic regression was used to estimate the risk of early losses, and mortality pre- and post-ART initiation, adjusting for relevant baseline covariates, including calendar period of enrollment which was included as a key variable of interest in this model. Post-hoc, relative goodness of fit of the logistic model was verified using a log-likelihood ratio to estimate a chi-square. Final models were checked using standard regression diagnostics for logistic regression. Wald confidence limits were used for all multivariate models.
- Our outcomes – pre-ART attrition from care, early losses, and mortality pre- and post-ART initiation– were determined at the outset and were not altered.
